# Thiamine and Micronutrient Deficiencies in Hospitalized Veterans Without Alcohol Use Disorder

**DOI:** 10.3390/diseases14030095

**Published:** 2026-03-05

**Authors:** Elisabeth A. Mates, Kellie Watkins, Christopher Sanchez, Nicolas Fiore, Claire Phibbs

**Affiliations:** 1 Department of Medicine, VA Sierra Nevada Healthcare System, Reno, NV 89502, USA; 2 Department of Research & Development, VA Sierra Nevada Healthcare System, Reno, NV 89502, USA; kelliewatkins@unr.edu (K.W.); christopher.sanchez2@ucsf.edu (C.S.); claire.phibbs@va.gov (C.P.); 3 School of Medicine, University of Nevada, Reno, Reno, NV 89557, USA; nicolas.fiore@unlv.edu; 4 Sierra Veterans Research and Education Foundation, Reno, NV 89502, USA

**Keywords:** thiamine, magnesium, cholecalciferol, folate, cobalamin, hospitalized, micronutrient deficiency

## Abstract

Background: Micronutrient deficiencies (MiDs) can increase medical complexity in hospitalized adults, but the prevalence in those without alcohol use disorder (AUD) is unknown. Our objectives were to prospectively determine the prevalence of thiamine, cobalamin, folate, magnesium, and cholecalciferol deficiencies in hospitalized veterans without AUD. Methods: Newly hospitalized veterans without AUD were recruited. Plasma thiamine, cholecalciferol, cyanocobalamin, folate, magnesium, C-reactive protein, albumin, and prealbumin were obtained. Interviews, physical exams, and medical record reviews were completed to assess clinical signs of MiDs, food insecurity, malnutrition, and hospitalization metrics. Pearson chi-square, Fisher’s exact, and logistic regression evaluated relationships among MiDs, malnutrition, food insecurity, demographics, and hospitalization metrics. Results: A total of 206 participants were enrolled, and 183 had partial results while 155 had complete results. The prevalences of deficiencies were 31.32% for magnesium, 27.07% for thiamine, 25.27% for cholecalciferol, 4.40% for cyanocobalamin, and 0.55% for folate. Malnutrition was reported by 50.60% of participants, and 56.00% reported food insecurity. Of those with biomarker MiDs, signs and symptoms were found in 97.92% with thiamine, 85.96% with magnesium, 67.39% with cholecalciferol, and 37.5% with cyanocobalamin deficiency. Cholecalciferol deficiency was associated with thiamine deficiency (*p* = 0.011, OR 3.180, CI 1.556–6.529), food insecurity (*p* = 0.0073, OR 2.690, CI 1.289–5.662), and longer length of hospital stay (*p* = 0.0401, IRR 1.295). All other associations were not statistically significant. Conclusions: In this exploratory pilot study, MiDs affected more than half of hospitalized veterans without AUD and were frequently associated with clinical signs and symptoms. TD affected one quarter of participants. Cholecalciferol deficiency was associated with longer hospital stays.

## 1. Introduction

The prevalence of micronutrient deficiencies (MiDs) in hospitalized veterans without alcohol use disorder (AUD) is not known but may be higher than the ambulatory population due to acute and chronic illnesses leading to hospitalization [1]. Hospital providers treating those with AUD, a common condition that predisposes patients to MiDs in high-income countries, will typically supplement patients with thiamine, folate, and multivitamins and routinely replace mineral deficiencies detected on blood chemistry analysis [2]. At our healthcare facility, which exclusively serves adult U.S. veterans, there are frequent hospital admissions for weakness, injuries sustained from ground-level falls, altered mental status, and adult failure to thrive (a combination of impaired physical functioning, malnutrition, depression, and cognitive impairment [3]). We hypothesized that thiamine deficiency (TD) or other common MiDs (such as folate, magnesium, cobalamin, and cholecalciferol) may contribute to those admissions but noted that there is minimal evidence regarding the prevalence of MiDs in those who do not have AUD. This study was conceived to fill this knowledge gap.

Thiamine is a key coenzyme in mitochondrial bioenergetics and is involved in the synthesis of molecules such as the neurotransmitters acetylcholine and γ-amino butyric acid, and various nucleotides [4]. Besides minuscule (<2%) amounts of thiamine produced by gut microbiota, the body’s supply must be continuously ingested due to limited body stores (25–30 mg) and constant consumption by metabolic processes [4,5]. Thiamine deficiency (TD) can develop quickly, after 18 days on a thiamine-deficient diet or within 72 h in critically ill patients [6,7]. TD causes a variety of signs and symptoms that have historically been described by four clinical syndromes: Wernicke’s encephalopathy (WE) characterized by a combination of two to four of encephalopathy, ophthalmoplegia, ataxia, and nutritional deficiency [7,8,9]; dry beriberi characterized by a combination of symmetric lower extremity muscle tenderness, loss of Achilles or patellar reflexes, weakness, and loss of sensation or paresthesia [4]; wet beriberi where signs of cardiac failure are present (some combination of tachycardia, peripheral or pulmonary edema, elevated central venous pressure, and hypotension) [4]; and gastrointestinal (GI) beriberi characterized by a combination of anorexia, nausea, vomiting, abdominal pain, constipation and lactic acidosis [10]. Overlapping clinical presentations are common in people with TD and are described by the term thiamine deficiency disorders (TDDs) [11]. The prevalence of TD in low- and middle-income countries (LMICs) where primary energy sources are low-thiamine staples such as polished white rice or cassava is hard to estimate because data are scarce in many regions but is as high as 35% in adults [12,13]. TD is thought to be rare in high-income countries [12], especially where thiamine fortification of food staples is routine, although prevalence data are lacking. Despite this, there are many published accounts of TD in high-income countries in acutely and chronically ill patients, such as those with end-stage renal disease (ESRD) [14], cancer [15], heart failure [16], dementia [17], acute psychiatric illness [18], stroke [19], diabetic ketoacidosis [20], critical illness [21], and medically complicated obesity [22]. There are no prevalence studies of TD in adults without AUD in a resource-rich country such as the United States (U.S.).

Magnesium is crucial for cell function because it is a cofactor for over 600 enzymes and is an activator for an additional 200 enzymes involved in cellular processes [23]. It also participates in DNA and RNA stabilization and repair, RNA transcription, and cardiac and nervous system function [23]. It is essential for healthy bone formation, calcium metabolism, activation of cholecalciferol, parathyroid hormone regulation, and glucose, lipid and protein metabolism, and is necessary for the functioning of the immune system [23,24]. Magnesium is necessary for the conversion of thiamine into its active form, and treatment of TD may be ineffective if concomitant magnesium deficiency is not corrected [9]. Adult body stores are about 25 g, with 1–2% in blood [23]. Changes in serum magnesium concentration in deficiency occur slowly, over months to years [25]. Signs of deficiency are not always present and can include concomitant hypocalcemia and hypokalemia, cardiac arrhythmia, carpo-pedal spasm, seizure, vertigo, ataxia, muscle cramps, lethargy, and weakness [24]. Magnesium deficiency has been associated with decreased bone mineral density and increased risk of fractures, hypertension, diabetes mellitus type 2, cancer, atherosclerotic vascular disease, neurological disease, and increased risk of death [23,24,26]. It is estimated that 45% of Americans are deficient, and 60% do not consume the recommended intake [27]. Despite magnesium’s importance in human health, there is a dearth of information on the prevalence of hypomagnesemia worldwide, probably because of the lack of a standardized laboratory test and because it is understudied compared to other micronutrients [27]. Reports of deficiency in hospitalized patients show prevalence ranging from 18 to 29% [26,28]. No studies have reported the prevalence of magnesium deficiency in hospitalized veterans without AUD.

Cholecalciferol is essential for the regulation of calcium and phosphorus metabolism and for bone mineral metabolism [29,30]. It has hormonal effects in cell nuclei, activating transcription for many genes, and the vitamin D receptor regulates the expression of over 900 genes [29]. In addition, it inhibits the expression of reactive oxygen species-generating factors such as NADPH oxidase [29]. The half-life of the active form of cholecalciferol (25-hydroxyvitamin D (25(OH)D)) is 20–30 days, but given the unpredictability of endogenous synthesis from sun exposure, it is difficult to predict how long it would take to become deficient if ingestion ceased [31]. A study in submariners who do not experience sun exposure found that tissue half-life is about 3 months [32]. Most individuals with deficiency are asymptomatic, but symptoms can include musculoskeletal pain, muscle spasm, weakness, and falls [29]. Deficiency resulting in bone demineralization causes rickets in children and osteomalacia in adults [29,30]. Deficiency has been linked to increased risk of fractures, susceptibility to autoimmune and infectious diseases, and reduced production of insulin [29]. The worldwide prevalence of cholecalciferol deficiency in adults ranges from 0% of males in Sri Lanka to 95.5% of females in Afghanistan [33]; however, for many LMICs there is no data available [30]. In one meta-analysis of cholecalciferol deficiency in US veterans, 40% were affected [34].

Folate acts as a cofactor in reactions known as one-carbon metabolism in amino acid and nucleotide metabolic pathways and is crucial for DNA and RNA synthesis and cell proliferation [35]. It is involved in methylation reactions such as the re-methylation of homocysteine to methionine and the methylation of DNA and histones in gene expression [35]. Total body stores of folate are 15–30 mg, which last 2–3 months on a deficient diet [35]. Signs and symptoms of deficiency are hard to specify because it is often concomitant with other nutritional deficiencies that cause symptoms, but can include megaloblastic anemia, oral and genital ulceration, and pancytopenia [5,35]. Deficiency has been linked to neural tube defects in pregnancy, cardiovascular disease, cognitive decline, and several forms of cancer [35]. Worldwide prevalence rates of folate deficiency are missing due to a lack of standardization of biomarker methodology, and surveys of deficiency are not always inclusive of the entire population [36]. In high-income countries, prevalence varies depending on food fortification practices ranging from < 1% in Canada to 13.4% in Israel [37,38]. There are no studies assessing the prevalence of folate deficiency in hospitalized U.S. veterans without AUD.

Cobalamin is a cofactor for methionine synthase that catalyzes the conversion of homocysteine to methionine, and it is the universal methyl donor for S-adenosylmethionine, which is necessary for methylation of 100 substrates including DNA, RNA, hormones, proteins, neurotransmitters, and phospholipids [5,39]. As such, cobalamin plays a role in the proliferation, maturation and regeneration of cells and is essential for the health of the nervous system. Total body stores are 2.5–5.0 mg, which can last 12–36 months with inadequate intake [5,39]. Signs of deficiency include macrocytic anemia, peripheral neuropathy, ataxia, spasticity, glossitis, mood impairment, and psychosis [39]. Subclinical deficiency is more common than clinical deficiency and is characterized by biomarker evidence of deficiency in the absence of symptoms [39]. As with folate, a lack of comprehensive worldwide survey data makes estimation of the prevalence of deficiency in LMICs difficult, but one review concluded there was no evidence that it was linked with the level of development [36]. In the US and Europe, rates of deficiency are 10–26% in adults [5] and as high as 40% in hospitalized elderly patients [40]. There are no prevalence studies on hospitalized veterans without AUD.

Systemic inflammation can affect plasma levels of vitamins due to redistribution of binding proteins and increased uptake by tissues [41]. An inflammatory response can be triggered by injury or illness, including infection, trauma, surgery, burns, cancer, pancreatitis and many acute and chronic diseases [42]. The degree of an inflammatory response can be measured by C-reactive protein (CRP) [42]. Inflammation leads to artificially decreased cholecalciferol levels when CRP is greater than 20 mg L^−1^ and artificially increased cobalamin levels when CRP is greater than 80 mg L^−1^ [41]. Inflammation does not appear to affect thiamine levels, and there is no evidence on how it affects folate [5]. There are two measurement techniques for CRP: standard and highly sensitive (hs-CRP). The hs-CRP assay was designed to detect low levels of inflammation that predict the risk of cardiovascular disease [43]. It is preferred to utilize standard CRP when assessing inflammatory impact on serum micronutrient levels [41]; however, there is good concordance between hs-CRP and CRP [43].

Our research objective was to determine the prevalence of TD in veterans without AUD who are admitted to the hospital. We also investigated the prevalence of cobalamin, folate, cholecalciferol, and magnesium deficiencies to determine if these deficiencies occur concomitant with TD and, if so, if they explain symptoms one could attribute to TD. Secondary objectives include the description of the clinical characteristics of adults with non-alcohol-related TD and the association among MiDs, malnutrition, and food insecurity. We hypothesized that MiDs, when combined with acute illness, increase medical comorbidity, which is associated with a higher risk of being admitted to a hospital, being readmitted after discharge, and having longer lengths of hospital stay (LOS) and higher mortality [44,45]. Our hypothesis was based on finding many cases of symptomatic TD in hospitalized veterans without AUD [46].

## 2. Materials and Methods

### 2.1. Ethics Statement

The study was approved by the University of Nevada, Reno Institutional Review Board (#1867369). We have adhered to the ethical principles of the Declaration of Helsinki. Written informed consent was obtained from each participant. The trial was registered prospectively with ClinicalTrials.gov: NCT05480943.

### 2.2. Study Population and Selection

This is an investigator-initiated observational prospective MiD prevalence pilot study of veterans aged 18 and older admitted to the medicine service at the Veterans Administration Sierra Nevada Healthcare System (VASNHCS) hospital from July 2022 to September 2024. All new admissions to the hospital were screened, and veterans were excluded if they reported excess alcohol intake (>14 drinks per week for men younger than 65 years old, or >7 per week for men 65 years or older or women, as defined by the National Institute of Alcohol Abuse and Alcoholism); were taking a B-complex vitamin or thiamine; or lived more than 70 miles from the hospital (a barrier to follow-up for a separate arm of the study). Those who passed the screening procedures were approached for consent. If they were unable to comprehend and “read back” basic elements of the informed consent (as determined by the U-ARE protocol [47]), they were not included due to prohibitive state laws.

We mitigated selection bias by screening all admissions to the medicine service without regard to admitting diagnosis, comorbid conditions, or demographics. A priori power calculations were completed and showed that a sample size of 290 would detect a prevalence of TD within +/− 5% of what we estimated to be 25% based on studies of comparable populations [48,49,50] with 95% confidence [51]. Due to unforeseen barriers to study procedures in this pilot study, our enrollment fell short of this goal.

### 2.3. Laboratory Procedures

Early morning fasting blood was drawn for measurement of thiamine, cobalamin, folate, cholecalciferol, complete blood count (CBC), prealbumin, and, for many participants, hs-CRP. Magnesium levels were obtained from the metabolic panel drawn on the day of admission (or as close to it as possible). Plasma thiamine was analyzed by Quest Diagnostics in Valencia, California USA using liquid chromatography/mass spectroscopy (LC-MS) with a normal range of 8–30 nmol L^−1^. CBC, hs-CRP, prealbumin, cholecalciferol, folate, magnesium, and cobalamin were measured locally at VASNHCS. Cholecalciferol (25(OH)D) was measured via chemiluminescence using the UniCel DxI Immunoassay System, with a normal range of 30–100 ng mL^−1^ (75–250 nmol L^−1^). For the purposes of this study, we chose a cutoff below 20 ng mL^−1^ (50 nmol L^−1^) as deficient per the recommendations of the Endocrine Society and Institute of Medicine [30]. Plasma folate was measured using the Beckman Coulter chemiluminescence competitive binding receptor assay, with a normal range of 2.8–20.0 ng mL^−1^ (6.34–45.31 nmol L^−1^). Plasma cobalamin was measured using the Beckman Coulter competitive binding immunoenzymatic assay, with a normal range of 180–1100 pg mL^−1^ (132–811 pmol L^−1^). Plasma magnesium was measured using a direct colorimetric method with a normal range of 1.8–2.5 mg dL^−1^. Plasma hs-CRP was measured using the Beckman Coulter AU 680 immune complex turbidometer (Beckman Coulter, Brea, CA, USA) with a normal range of 0.02–0.75 mg dL^−1^. Prealbumin was measured using the Beckman Coulter AU 680 immune complex turbidometer with a normal range of 18–38 mg dL^−1^. CBC and mean corpuscular volume (MCV) were measured using the Sysmex Corporation XN-3100 hematology analyzer (Sysmex Corporation, Hyogo, Japan) with a normal hemoglobin (Hg) range of 14.0–18.0 g dL^−1^ for males and 12.0–16.0 g dL^−1^ for females, a normal hematocrit of 38.0–51.0% for males and 36.0–47.0% for females, and a normal MCV of 85.0–100.0 femtoliter (fL) for both.

### 2.4. Patient Interview, Chart Extraction, and Examination

Participants were interviewed for symptoms of TDDs, including weakness, falling, double vision, confusion, memory loss, hallucinations, seizures, anorexia, nausea, vomiting, constipation, and imbalance. We enquired about food insecurity using responses to three statements: “I worried my food would run out before I got money to buy more”, “the food that I bought just didn’t last and I didn’t have money to get more”, and “I depend on donations for food such as at the homeless shelter or a food pantry”. Responses could be never true (score 0), sometimes true (score 1), or often true (score 2). We added points for each answer and considered a score above zero indicative of food insecurity (range 0–6). We asked about alcohol intake and categorized them as none for one drink or less per month, mild for less than 4 drinks a week, or moderate for 3 to 7 drinks a week for women and men aged 65 and older or more than 3 but less than 14 a week for men younger than 65.

Standardized physical exams were carried out by E.A.M. or one of four trained sub-investigators late in the hospital stay after the medical condition for which they were hospitalized improved. Vital signs, including height, weight, and body mass index (BMI) at the time of their physical assessment, were recorded. Mental status was assessed using the Montreal Cognitive Assessment (MOCA). Delirium was assessed using the 4AT test. Extraocular movement testing was carried out and disconjugate gaze or nystagmus noted. Motor strength was assessed for shoulder abduction, arm flexion and extension, hip flexion, knee extension, dorsiflexion and plantar flexion using a scale from zero to five, where zero was no movement, one was visible muscle contraction but no movement, two was movement but not against gravity, three was movement against gravity, four was able to resist the examiner somewhat but gives way, and five was able to resist examiner without giving way. Anything less than five was considered weak. Cerebellar function was assessed using the finger-to-nose test in the upper extremities, the heel-to-shin test in the lower extremities, and gait observation. Auscultation was used to identify rales on lung exam consistent with pulmonary edema, cardiac rhythm and any extra heart sounds, and the presence or absence of bowel tones. Perception of light touch on the extremities was assessed for the presence of neuropathic symptoms, and we examined for the presence of pitting edema. Lastly, an exam for signs of malnutrition included the presence or absence of temporal wasting, orbital or supraclavicular fat pad loss, scapular or rib prominence, and hand interosseus muscle loss. Practitioners were not blinded to vitamin level results, although most were not available at the time of examination due to delays in processing.

Most participants had received care at our facility previously, and weights were evaluated over the preceding year to determine the percentage of weight loss or gain. If weight history was not available but participants reported weight loss, 5% weight loss in the preceding 6 months was assigned. We used the Global Leadership Initiative on Malnutrition (GLIM) criteria [52] to determine whether malnutrition was present. Phenotypic criteria included percentage weight loss of more than 5% in the preceding 6 months, low BMI (<20 for age < 70 or <22 for age ≥ 70), or reduced muscle mass (determined by examiners as having 2 or more physical signs of malnutrition). Etiologic criteria were reduced food intake as reported by the participants or the presence of inflammation determined by elevated hs-CRP or low prealbumin. If an individual had at least one each of the phenotypic and etiologic criteria, they were classified as having malnutrition.

A systematic review of each participant’s electronic medical record was carried out by E.A.M. or one of the five trained associates. Basic demographics at enrollment were recorded. Chronic medical conditions existing prior to admission were extracted. Acute medical conditions identified by the hospital care team were recorded in addition to symptoms reported by participants. Outpatient medications and supplement use prior to hospital admission were recorded, and medication refill history was confirmed. A medication or supplement was not included if it had not been refilled in the preceding month, ensuring an adequate supply. Supplements prescribed by or purchased at non-VA locations were included, but refill history could not be confirmed. Hospital LOS, readmission within 30 days of hospital discharge, and death within 90 days of hospitalization were recorded. For three participants with very long LOS due entirely to social barriers to discharge, LOS was adjusted down to the number of days until the acute medical illness was resolved.

After interview and examination, it was determined whether an individual met the criteria for one of the clinical syndromes associated with TD: WE, wet beriberi, dry beriberi, or GI beriberi. WE was determined using the Caine criteria [8], namely the presence of two of the following: malnutrition, abnormal cerebellar function, ophthalmoplegia (either disconjugate gaze or nystagmus), or encephalopathy (either delirium by the 4AT test, low MOCA score, or hallucinations). Wet beriberi was defined as pulmonary or peripheral edema with no alternate explanation. Conditions considered plausible alternate explanations were prior diagnosis of heart failure, ischemic cardiomyopathy, valvular heart disease, venous insufficiency, chronic kidney disease stage IV or higher, nephrotic syndrome, chronic loop diuretic use, bacterial or viral pneumonia causing lung rales, pulmonary fibrosis, rheumatologic lung disease, severe sepsis requiring large volume fluid resuscitation, and use of medications known to cause peripheral edema (e.g., amlodipine, abiraterone). Dry beriberi was defined as motor weakness of more than 2 muscle groups or acute-onset peripheral neuropathy not explained by pre-existing conditions (e.g., spinal stenosis, stroke, multiple sclerosis, diabetic neuropathy) or chronic use of medications to treat neuropathy (e.g., gabapentin, pregabalin). GI beriberi was defined as symptoms of anorexia, nausea, vomiting, constipation, or radiologic evidence of ileus or constipation in the absence of an alternative explanation. Examples of alternative explanations include infectious gastroenteritis, bowel obstruction, pancreatitis, GI hemorrhage, colitis, intra-abdominal or retroperitoneal infection, nephrolithiasis, and intra-abdominal thrombosis.

### 2.5. Statistical Analysis

All measurements were taken and reported once for each participant. The prevalence of MiDs was calculated as (patients with the MiD)/(patients in the cohort with an MiD result). Measures of association between categorical variables were assessed using the Pearson chi-squared test, as well as respective odds ratios and 95% confidence intervals. If 25% of cells had expected counts of less than five, Fisher’s exact replaced the Pearson chi-square. Statistical significance was determined by a two-sided *p*-value < 0.05. Missing values were excluded from analyses because they were ≤ 10% of the sample composition. Logistic regression assessed relationships among each MiD, malnutrition, and food insecurity, and between alcohol consumption and TD, with the associated odds ratios and 95% confidence intervals reported per level of interest. Logistic regression analyses were univariate models given the limited sample size. Negative binomial regression adjusting for age and sex tested associations between LOS and micronutrient deficiencies because LOS was over-dispersed. Wilcoxon rank sum compared TD symptom and clinical syndrome counts between those with TD and those with both TD and magnesium deficiencies. All analyses were conducted using RStudio 2024.09 and R 4.4.1 on the Veterans Affairs Informatics and Computing Infrastructure (VINCI) and Microsoft Excel for Microsoft 365 MSO version 2408.

## 3. Results

### 3.1. Participants

**Figure 1:**
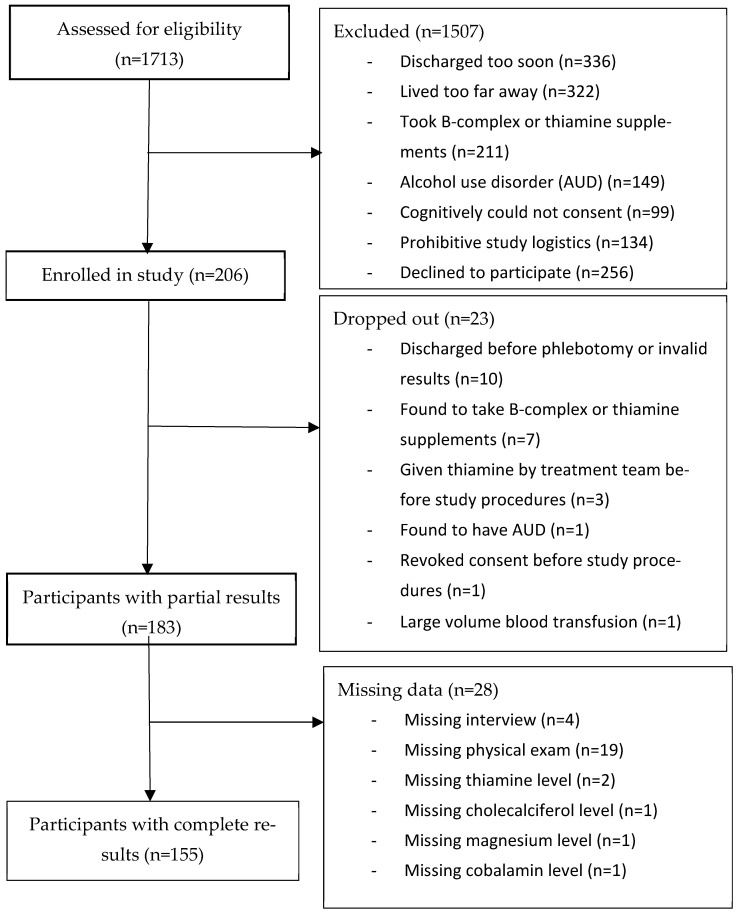
Flowchart of participants.

Figure 1 illustrates the flow of participants in the study. The number of potential participants screened for the study was 1713. The number enrolled was 206. Of those, 183 had partial results and 155 had complete results.

### 3.2. Prevalence of Micronutrient Deficiencies, Malnutrition, and Associations

Table 1 shows the characteristics of the participants versus the prevalence of MiDs. The average age was 70.64 years, and the majority of participants were white non-Hispanic males, reflective of our inpatient population. Among participants, 31.32% with magnesium results were deficient, 27.07% with thiamine results were deficient, 25.27% with cholecalciferol results were deficient, 8 of 182 with cobalamin results were deficient (4.40%), and 1 of 183 with folate results were deficient (0.55%). Overall, 105/183 (57.38%) had at least one MiD, and 44/183 (24.04%) had more than one deficiency. There was a statistically significant relationship between age and cholecalciferol deficiency, with patients aged 60–69 having the greatest observed deficiency rate.

Table 2 shows associations among thiamine, cholecalciferol, and magnesium deficiencies. TD was correlated with cholecalciferol deficiency (*p* = 0.0011, odds ratio (OR) 3.180, 95% confidence interval (CI) 1.556–6.529). For the subset of participants with available hs-CRP data (n = 122), this association was maintained when controlling for hs-CRP (O.R. 3.50, 95% CI 1.442–8.631). Due to the infrequency of abnormal results, associations between cyanocobalamin and folate deficiencies and any other MiD could not be statistically evaluated, but of those with cobalamin deficiency four of eight (50.00%) also had TD, and three of eight (37.50%) had concomitant cholecalciferol deficiency. The one individual with folic acid deficiency also had magnesium and thiamine deficiencies.

We did not find any statistical relationship between thiamine or magnesium deficiencies and high hs-CRP. Of the 46 participants who had low cholecalciferol levels, 30 had hs-CRP results, of which 15 (50.00%) had hs-CRP levels greater than 20 mg L^−1^, which has been associated with artificially low levels [41]. Of the 182 with cobalamin results, 125 had hs-CRP measurements, of which 33 (26.40%) were greater than 80 mg L^−1^, which has been associated with artificially elevated levels [41]. Cobalamin and folate deficiencies could not be statistically evaluated due to the low frequency of abnormal results.

Table 3 shows associations among malnutrition, food insecurity and thiamine, cholecalciferol, and magnesium deficiencies. There were 168 participants with enough data to determine malnutrition using the GLIM criteria, of which 85 (50.60%) had malnutrition. There were 168 participants who responded to the food insecurity survey, of which 56 (33.33%) reported food insecurity. There were no statistically significant associations between malnutrition and any MiD. Food insecurity was associated with cholecalciferol deficiency (*p* = 0.0073, OR 2.690, CI 1.289–5.662). Cobalamin and folate deficiencies could not be statistically evaluated due to the low frequency of abnormal results; however, none with cobalamin deficiency reported food insecurity, and two of seven with malnutrition assessments had malnutrition (28.57%). The one with folate deficiency did not report food insecurity but did have malnutrition.

Table 4 shows relationships among thiamine, cholecalciferol, and magnesium deficiencies with markers of higher medical comorbidity: hospital LOS, death within 90 days of discharge, and hospital readmission within 30 days. Thiamine and magnesium deficiencies were not associated with these metrics. Cholecalciferol was associated with longer LOS (*p* = 0.0401, IRR 1.295, CI 1.012–1.657).

Table 5 shows the relationship between TD and alcohol use among participants. There was no significant relationship between no, mild, or moderate alcohol consumption and TD.

### 3.3. Micronutrient Deficiency Signs and Symptoms

Table 6 shows symptoms of TD reported during the interview and clinical TD syndromes diagnosed by study providers for those found to have biomarker TD. Of the 49 participants with plasma thiamine less than 8 nmol L^−1^, six were missing exams and one was missing an exam and an interview. Thirty-nine had symptoms and a syndrome, three had symptoms without meeting criteria for a syndrome, five had symptoms but were missing exams, and one had no symptoms or clinical syndromes. The most common symptoms were loss of appetite (42/48, 87.50%), loss of balance (30/48, 62.50%), nausea or vomiting (29/48, 60.42%), constipation (28/48, 58.33%), weakness or falling (26/48, 54.17%), confusion or trouble thinking (23/48, 47.92%), and memory loss (17/48, 35.42%). Less common symptoms included double vision (6/48, 12.50%) and hallucinations (3/48, 6.25%), and none had reported or witnessed seizures. Of the clinical syndromes of TD, 26/43 (60.47%) met criteria for Wernicke’s encephalopathy, 29/48 (60.42%) for GI beriberi, 25/43 (58.14%) for dry beriberi, and 3/43 (6.98%) for wet beriberi. Four had no syndrome, and more than half had two or more TD syndromes, with sixteen having two, nine having three, and three having all four syndromes. Of those meeting criteria for Wernicke’s encephalopathy based on malnutrition and ataxia, two had concomitant magnesium deficiency. Of those meeting criteria for dry beriberi based on motor weakness, seven had concomitant magnesium deficiency, five had cholecalciferol deficiency, and four had all three deficiencies. Comparing those with isolated TD to those with concomitant thiamine and magnesium deficiencies using Wilcoxon Rank Sum, those with both deficiencies reported more symptoms of TD (*p* = 0.0032, OR 1.655, CI 1.195–2.527) but did not have a statistically significant increase in the number of clinical syndromes (*p* = 0.2313, OR 1.287, CI 0.777–2.193).

Of the 179 participants who completed surveys, only 10 (5.59%) reported no symptoms of TD, three of whom had low plasma thiamine. Of the 164 who completed exams, only 24 (14.63%) did not meet criteria for a TD syndrome, four of whom had low plasma thiamine.

Of the 46 participants with cholecalciferol deficiency, 31 (67.39%) had signs or symptoms of deficiency with 26 reporting weakness or falling (56.52%) and 20 (43.48%) having motor weakness on exam (14 having concomitant magnesium or thiamine deficiency). None were given a diagnosis of falling by inpatient providers or were admitted with a fracture. We did not ask whether they had muscle pain. Of the 57 with magnesium deficiency, 49 (85.96%) had signs or symptoms, of which 38 complained of weakness or falling (66.67%), 28 (49.12%) had motor weakness on exam (16 having concomitant cholecalciferol or thiamine deficiency), 10 (17.54%) had ataxia in at least one limb or gait (of which four had concomitant TD), four (7.02%) had an arrhythmia (atrial fibrillation, supraventricular or ventricular tachycardia, or torsade de point), and none had vertigo or seizure. One reported muscle cramps to the hospital provider, but we did not ask about this symptom or whether they had carpo-pedal spasm or lethargy. Of the eight with cobalamin deficiency, 37.50% were symptomatic, one had new onset peripheral neuropathy, one had ataxia without an alternative explanation, one complained of hallucinations but also had TD, and none had macrocytic red blood cell indices. We did not assess for spasticity, glossitis, or mood impairment. The one patient with folate deficiency had macrocytic anemia, and we did not ask them about oral or genital ulceration.

### 3.4. Participant Use of Supplements Prior to Hospitalization

Of the 183 participants, 57 (31.15%) were taking cyanocobalamin before hospitalization, none of whom were deficient. A total of 86 (47.00%) were prescribed cholecalciferol, of whom nine were deficient, and 41 (22.40%) were prescribed magnesium, of whom 20 were deficient. Seven (3.83%) were prescribed folate, of whom none were deficient. As per study protocol exclusions, none were prescribed thiamine or vitamin B-complex. Fifty-nine (32.24%) were taking a multivitamin, of which none were cobalamin deficient, one was folate deficient, seven were cholecalciferol deficient, nine were thiamine deficient, and 20 were magnesium deficient. Twenty-one were prescribed an oral nutrition supplement, of which one was cobalamin deficient, four were cholecalciferol deficient, seven were thiamine deficient, and seven were magnesium deficient.

## 4. Discussion

This is the first prevalence study of TD and MiDs in hospitalized veterans without AUD, addressing a knowledge gap. More than half had at least one MiD, and many were symptomatic. Magnesium was the most common deficiency (31.32%) and probably underestimated because plasma levels represent less than 1% of total body stores [23]. TD was surprisingly common (27.07%) given the lack of AUD, and TD is reportedly rare in high-income countries [7,12]. TD was not statistically associated with mild or moderate alcohol use. We found, as expected, a prevalence of folate deficiency (0.5%) and a lower-than-expected prevalence of cholecalciferol (25.3%) and cyanocobalamin (4.4%) deficiencies, likely due to supplement use prior to admission. It is possible the number with cyanocobalamin deficiency was underestimated because of the presence of inflammation, which can artificially elevate blood levels [41]. The prevalence of cholecalciferol deficiency may be overestimated because we did not adjust for concomitant inflammation [5] due to many missing hs-CRP measurements. The rate of malnutrition in our study was 51%, which is consistent with similar studies using GLIM criteria, but it was not correlated with any MiD. It is possible that malnutrition was not correlated with TD because those with moderate to severe malnutrition may have been prescribed thiamine by their treatment team, making them ineligible for our study. In addition, our lower-than-planned enrollment may have led to underpowering of this assessment. Food insecurity was associated with cholecalciferol deficiency but not with other MiDs. We did not find that thiamine or magnesium deficiencies were correlated with LOS, death within 90 days, or hospital readmission; however, cholecalciferol deficiency was associated with longer LOS. We expected TD would be associated with these metrics given the number of participants affected by WE and dry beriberi, which cause debility, but low enrollment and underpowering could explain this.

Nearly all participants with TD (97.92%), 85.96% with magnesium deficiency, and 67.39% with cholecalciferol deficiency had signs or symptoms that could be attributed to the MiD. Fewer with cobalamin deficiency were symptomatic (37.50%), probably indicating that the majority had subclinical deficiency. The one veteran with folate deficiency had macrocytic anemia. Due to the limitations of the study design, the percentage of those with symptoms of MiDs other than thiamine was likely underestimated because we did not ask about all symptoms of each deficiency. Perceived and actual muscle weakness was the most common manifestation of magnesium and cholecalciferol deficiencies. Symptoms of TD were more frequent in those with concomitant magnesium deficiency, probably due to biochemical interdependence: magnesium is a necessary cofactor for thiamine activation and several thiamine-dependent enzymatic pathways [9]. Of the TD clinical syndromes, Wernicke’s encephalopathy and GI beriberi were the most common, followed by dry beriberi. Wet beriberi was uncommon but probably underestimated because if any other acute or chronic diagnosis could explain the clinical findings, we did not classify them as having wet beriberi. Most TD cases where a clinical exam was completed had more than one TD syndrome (65.12%), as previously described [11], highlighting the fact that TD causes multisystem dysfunction. Because of confounding factors such as concomitant MiDs and underlying medical conditions, we cannot be certain that the signs and symptoms are a result of a specific MiD.

It is unclear why so many hospitalized veterans have TD, since thiamine is present in common food sources such as fortified cereal, wheat flour, and pork. Causes of TD can be due to insufficient intake (low consumption, malabsorption, or toxin interference), increased consumption of thiamine stores (in critical illness or high carbohydrate diets) and increased losses (due to vomiting, diarrhea, dialysis, or chronic diuretic use) [46]. One possibility is that thiamine is heat labile, and cooking breaks down up to 30% of the vitamin [4]. Other potential causes include poor appetite with reduced intake due to illness in the weeks prior to hospitalization and increased metabolic demands due to acute illness [5]. Future studies of TD should include detailed food intake surveys and an analysis of specific causes of accelerated thiamine consumption or increased losses.

Previous studies of TD in high-income countries have focused on adults with specific diagnoses such as heart failure [16], stroke [19], diabetic ketoacidosis [20], obesity [22], and end-stage renal disease [14] for example. There are a few prevalence studies in hospitalized adults without specific diagnoses that are comparable to our study, but none exclude those with AUD. O’Keeffe et al. report 17% definite and 31% marginal cases of TD in hospitalized elderly in Ireland using the erythrocyte transketolase assay (ETKA) biomarker [53]. Lee et al. found a 14% prevalence of TD in elderly adults presenting to a U.S. emergency department with low plasma thiamine levels [50]. Pepersack et al. showed 39% moderate and 5% severe TD by ETKA in elderly adults admitted to the hospital in Belgium [54]. Lemoine et al. found that 25% of hospitalized adults in France had TD by ETKA [48]. Direct comparison of studies of TD is difficult due to a lack of commonly agreed-upon gold standard biomarkers [12]. Our results combined with these studies contradict the assertion that TD is rare in high-income countries. Our data show it is common in veterans without AUD who are admitted to the hospital.

It is debatable how to accurately define cases of TD: by biomarker level, suggestive clinical picture, thiamine-responsive symptoms, or a combination of these factors [11,12]. Symptoms of TD are non-specific and are suffered by many adults without TD, making diagnosis solely on clinical grounds overly sensitive. In our study, only 5.59% lacked any symptoms, and only 14.63% did not meet criteria for a TD syndrome. We elected to identify cases of TD by low plasma thiamine levels supported by clinical findings. All but one individual identified in this manner had clinical findings explainable by TD. Defining TD by biomarker alone is problematic due to a lack of a gold standard with a commonly agreed-upon reference range [12]. The historical functional blood test, ETKA, is no longer commercially available and has been supplanted by direct measurement of thiamine and its phosphate esters in plasma or whole blood [12]. It has been argued that whole blood thiamine (primarily made up of thiamine diphosphate (TDP) in red blood cells (RBCs)) is superior to plasma thiamine (primarily free thiamine and thiamine monophosphate) [12]; however, our study shows excellent concordance between low plasma thiamine and clinical TD. There are several reasons why whole blood TDP may not be a good biomarker of clinical TD. First, cellular bioenergetics that consume TDP in its role as cofactor are different in RBCs (which lack nuclei and mitochondria) than in nucleated cells elsewhere in the body [55]. Of the four enzyme systems utilizing TDP, three (the pyruvate dehydrogenase complex, the branched chain α-ketoacid dehydrogenase complex, and the α-ketoglutarate dehydrogenase complex) are absent in RBCs, and the fourth, transketolase, has lower activity in RBCs than in other cells [56], so it is reasonable to assume that RBCs consume thiamine at a slower rate. Warnock et al. showed that the rate of depletion of TDP in RBCs in rats on a thiamine-deficient diet was 5–6 days slower than in the liver, heart, and brain [57]. Chen et al. showed that in rats on a thiamine-deficient diet, plasma thiamine drops by 88% on day 5, whereas RBC TDP takes 17 days to drop 81% [58]. Nath et al. showed that only 23/66 patients with clinical TD had low whole blood thiamine levels [22]. These studies provide evidence that whole blood TDP is not representative of brain, gut, or other organ system thiamine levels.

One drawback to the current measurement techniques for thiamine biomarkers is the need to ship frozen specimens to reference laboratories with LC-MS capability, resulting in a 7- to 10-day delay in obtaining results. This presents a barrier to rapid diagnosis and treatment of TDDs, especially when hospital systems encourage prompt discharge of patients. Our results, showing high prevalence of symptomatic TD, support the recommendation to replete thiamine when deficiency is clinically suspected and not order or wait for biomarker results, especially in patients admitted to an intensive care unit, those with AUD, or those with refeeding syndrome [5,59]. Thiamine is inexpensive and has an excellent safety profile, making empiric treatment low risk [60]. As such, it is important for clinicians to be familiar with the clinical syndromes of TD. The creation and validation of a clinical scoring system for decision support and for the development of a rapid, inexpensive test that can be run locally would both be invaluable tools. The technique described by Edwards et al. is a promising candidate for rapid point-of-care biomarker assessment [61].

Cholecalciferol deficiency was common in our cohort and correlated with prolonged length of hospital stay, which is consistent with prior studies [62,63] indicating it contributes to medical comorbidity. There are many potential explanations for this, given cholecalciferol’s effects on the immune and cardiovascular systems and its modulating effects on hundreds of genes [29,62]. Observational studies have shown that cholecalciferol deficiency is linked to the development of sepsis, bloodstream infections, respiratory illness, and increased cost of intensive care unit stays [62,63]. On the other hand, supplementation studies in hospitalized patients have not shown statistically significant benefits to mortality or LOS except in those with severe deficiency, where supplementation resulted in lower mortality [62,63]. The heterogeneity of assays and reference ranges used to assess cholecalciferol status and the dosage and duration of supplementation in various studies may explain the lack of effect on outcomes [63]. We found that cholecalciferol deficiency was correlated with food insecurity, suggesting it may be a marker of broader nutritional and social vulnerability. Our use of immunoassay to assess plasma cholecalciferol levels is subject to error due to cross-reactivity with similar metabolites [64], limiting the generalizability of our results. Due to incomplete data on hs-CRP, we did not control for it in the analyses of cholecalciferol deficiency associations. We did explore stratified and sensitivity analyses for the correlation between cholecalciferol and thiamine deficiencies for 122 participants with hs-CRP and did not find that it altered results in a meaningful way.

Magnesium deficiency has been linked with clinical disease affecting nearly every organ system [23], and other studies have shown that it is associated with increased risk of mortality and prolonged LOS [65]. Our population’s older age and consumption of the typical US Western diet put them at higher risk for hypomagnesemia [23], possibly explaining the high prevalence in our sample. Fiorentini et al. assert that chronic hypomagnesemia is associated with or worsens many diseases, such as diabetes, osteoporosis, and cardiovascular disease, increasing the cost of healthcare [23]. Of our participants, 41 were prescribed magnesium prior to enrollment, 20 of whom were still deficient, indicating that a significant portion had chronic hypomagnesemia. We did not find that hypomagnesemia was associated with 90-day mortality, readmission, or hospital LOS, although many had signs or symptoms of deficiency. The lack of association with markers of comorbidity is most likely due to low enrollment and the study was not powered to detect these effects. Less than 1% of the total body magnesium is present in blood, and measurement of serum magnesium does not necessarily reflect total body stores [27]. In addition, serum levels can change hourly in response to dietary intake, excretion in the kidneys, and equilibration with tissue stores [27], another potential explanation for the lack of correlation with markers of comorbidity. On the other hand, screening for and treating hypomagnesemia is inexpensive [23] and low-risk and should be considered in hospitalized veterans.

The strengths of this study include the concomitant evaluation of malnutrition, micronutrient biomarkers, and clinical evaluation for signs of MiDs. We explored the relationships between concomitant MiDs hoping to find a surrogate marker for TD, since the time required to obtain biomarker results leads to diagnostic uncertainty and potential delays in treatment. We found that thiamine and cholecalciferol deficiencies were correlated, although it is unclear why. Neither deficiency was associated with malnutrition, and overlap of dietary sources and mechanisms of absorption is minimal, so we do not suggest using cholecalciferol as a surrogate marker for TD. Some with symptomatic TD had concomitant magnesium, cholecalciferol, and cobalamin deficiencies, which could explain their symptoms; however, due to low numbers, we were unable to statistically evaluate which deficiency was more likely to explain symptoms.

Limitations of the study include that this is an exploratory pilot study with a small sample size falling short of our enrollment goal. As such, it was underpowered, limiting our ability to provide generalizable results, find relationships among risk factors, and control for potential confounders. Those taking thiamine or B-complex supplements were excluded, which could have removed individuals at high-risk, influencing prevalence estimates and associations with malnutrition. The generalizability of our results and ability to detect trends in demographic characteristics were further limited by the fact our cohort was composed of primarily older, white, non-Hispanic males limited to a single center. We lacked food intake surveys, which may have provided insight into the dietary factors influencing MiDs. There were confounding factors not accounted for, including but not limited to socioeconomic status, comorbid medical conditions, substance use other than alcohol, and dietary patterns. There was a potential for misclassification, as we categorized several continuous or re-categorized rank-order variables based on either clinical practice or to overcome sample-size limitations. Future studies of larger samples with generalizable populations are needed to corroborate our findings, control for confounding variables, and provide sufficient data to confirm the true prevalence of MiDs among veterans without AUD.

## 5. Conclusions

In this exploratory pilot study, we found MiDs were common, affecting more than half of hospitalized veterans without AUD, many of whom were symptomatic. TD was not rare, affecting one quarter of participants, with WE and GI beriberi being the most common TD syndromes followed by dry beriberi. Malnutrition was common (50.60%) but was not associated with MiDs. Food insecurity was common (33.33%) and associated with cholecalciferol deficiency. In the case of TD, treatment should be started in those with suspicious symptoms to avoid therapeutic delays while waiting for biomarker confirmation. Further study, including larger numbers and more diverse participants, would be valuable for validating these findings.

## Figures and Tables

**Table 1:** Micronutrient deficiencies by demographics for 183 participants.

	Micronutrient Deficiencies					
	Thiamine (n = 49)		Cholecalciferol (n = 46)		Magnesium (n = 57)	
	n (%)	*p*-Value	n (%)	*p*-Value	n (%)	*p*-Value
**Age**						
<50 (N = 11)	5 (45.45%)	0.1626	4 (36.36%)	<0.0001	2 (18.18%)	0.4032
50–59 (N = 11)	3 (27.27%)		3 (27.27%)		3 (27.27%)	
60–69 (N = 47)	16 (34.04%)		23 (48.94%)		20 (42.55%)	
70–79 (N = 83)	21 (25.30%)		15 (18.07%)		24 (28.92%)	
>80 (N = 31)	4 (12.90%)		1 (3.23%)		8 (25.81%)	
**Birth Sex**						
Male (N = 175)	45 (25.71%)	0.2149	44 (25.14%)	1.0000	54 (30.86%)	0.7069
Female (N = 8)	4 (50.00%)		2 (1.14%)		3 (37.50%)	
**Race**						
White (N = 159)	45 (28.30%)	0.7620	38 (23.90%)	0.2510	51 (32.08%)	0.3909
Black (N = 9)	3 (33.33%)		4 (44.44%)		3 (33.33%)	
Asian (N = 2)	0 (0.00%)		0 (0.00%)		0 (0.00%)	
AIAN (N = 4)	0 (0.00%)		1 (25.00%)		0 (0.00%)	
NHPI (N = 1)	0 (0.00%)		1 (100.00%)		1 (100.00%)	
Unknown (N = 8)	1 (12.5%)		2 (25.00%)		2 (25.00%)	
**Ethnicity**						
Hispanic (N = 6)	1 (16.67%)	1.0000	1 (16.67%)	1.0000	0 (0.00%)	0.1787
Non-Hispanic (N = 173)	48 (27.75%)		45 (26.01%)		56 (32.37%)	
Unknown (N = 4)	0 (0.00%)		0 (0.00%)		1 (25.55%)	

N is the number of participants per category, and n is the number with deficiency per category. Missing data were excluded from analyses due to ≤10% of sample composition. Fisher’s exact tested associations due to 25% of expected cell frequencies < 5, with *p* ≤ 0.05 considered significant except for associations with age, where expected cell frequencies were 20%. Cyanocobalamin and folate were omitted due to low counts. AIAN = American Indian/Alaskan Native; NHPI = Native Hawaiian/Pacific Islander.

**Table 2:** Comorbidity of micronutrient deficiencies (N = 183).

	Micronutrient Deficiencies			
	n (%)	*p*-Value	Odds Ratio	95% CI
**Thiamine Deficiency (n = 49)**				
**Cholecalciferol**				
Deficient (N = 46)	21 (45.65%)	0.0011	3.180	1.556–6.529
Normal (N = 136)	28 (20.59%)		Ref	Ref
Missing (N = 1)	0 (0.00%)		––	––
**Magnesium**				
Deficient (N = 57)	19 (33.33%)	0.2099	1.550	0.773–3.074
Normal (N = 125)	30 (24.00%)		Ref	Ref
Missing (N = 1)	0 (0.00%)		––	––
**Cholecalciferol Deficiency (n = 46)**				
**Magnesium**				
Deficient (N = 57)	17 (29.82%)	0.3555	1.392	0.681–2.798
Normal (N = 125)	29 (23.20%)		Ref	Ref
Missing (N = 1)	0 (0.00%)		––	––

Each micronutrient per other micronutrient is depicted as n/N, where n is the total number with the micronutrient deficiency per column and N is the total number per row. C.I. = confidence interval. Chi-square analysis tested associations, with *p* ≤ 0.05 considered significant. Logistic regression produced odds ratios with significance assessed using 95% confidence intervals. Reference groups (Ref) for each micronutrient were not deficient/normal. Double dashes indicate that analyses excluded missing values due to low counts (n ≤ 2; ≤10% total sample). Cobalamin and folate were not included due to low frequency of abnormal results.

**Table 3:** Associations among malnutrition, food insecurity, and micronutrient deficiencies for N = 183 participants.

	Micronutrient Deficiency			
	Thiamine (n = 49)			
	n (%)	*p*-Value	Odds Ratio	95% CI
**Malnutrition**				
Yes (N = 85)	24 (28.24%)		1.531	0.753–3.169
No (N = 81)	17 (20.99%)	0.2411	Ref	Ref
Unknown (N = 17)	8 (47.06%)		––	––
**Food Insecurity**				
Yes (N = 56)	13 (23.21%)	0.4930	0.770	0.356–1.600
No (N = 112)	31 (27.68%)		Ref	Ref
Unknown (N = 15)	5 (33.33%)		––	––
	**Cholecalciferol (n = 46)**			
	n (%)	*p*-value	Odds Ratio	95% CI
**Malnutrition**				
Yes (N = 85)	23 (27.06%)		1.278	0.630–2.624
No (N = 81)	18 (22.22%)	0.4982	Ref	Ref
Unknown (N = 17)	5 (29.41%)		––	––
**Food Insecurity**				
Yes (N = 56)	20 (35.71%)	0.0073	2.690	1.289–5.662
No (N = 112)	19 (16.96%)		Ref	Ref
Unknown (N = 15)	7 (46.67%)		––	––
	**Magnesium (n = 57)**			
	n (%)	*p*-value	Odds Ratio	95% CI
**Malnutrition**				
Yes (N = 85)	27 (31.76%)		1.154	0.593–2.256
No (N = 81)	23 (28.40%)	0.6737	Ref	Ref
Unknown (N = 17)	7 (41.18%)		––	––
**Food Insecurity**				
Yes (N = 56)	15 (26.79%)	0.5273	0.794	0.381–1.603
No (N = 112)	35 (31.25%)		Ref	Ref
Unknown (N = 15)	7 (46.67%)		––	––

For n/N, n is the total number with the micronutrient deficiency per column, and N is the total number per row. Chi-square analysis tested associations with *p* ≤ 0.05 considered significant. Logistic regression produced odds ratios with significance assessed using 95% confidence intervals (C.I.). Analyses excluded missing values due to low counts (≤10% missing in the total sample). Reference groups (Ref) for each micronutrient were not deficient/normal. Double dashes indicate analyses excluded missing values due to low counts (n ≤ 10% total sample).

**Table 4:** Associations among micronutrient deficiencies, malnutrition, and indicators of medical comorbidity for N = 183 participants.

	Thiamine (n = 49)			
	n (%)	*p*-Value	IRR/Odds Ratio	95% CI
**Length of Stay (days)**				
	––	0.9616	0.994	0.781–1.262
**Death within 90 days**				
Yes (N = 23)	5 (21.74%)	0.6556	0.787	0.247–2.130
No (N = 159)	43 (27.04%)		Ref	Ref
Unknown (N = 1)	1 (100.00%)		––	––
**Hospital Readmission within 30 days**				
Yes (N = 28)	7 (25.00%)	0.8845	0.933	0.345–2.279
No (N = 155)	42 (27.10%)		Ref	Ref
Unknown (N = 0)	0 (0.00%)		––	––
	**Cholecalciferol (n = 46)**			
**Length of Stay (days)**				
	––	0.0408	1.295	1.012–1.657
**Death within 90 days**				
Yes (N = 23)	4 (17.39%)		0.581	0.162–1.655
No (N = 159)	42 (26.42%)	0.3442	Ref	Ref
Unknown (N = 1)	0 (0.00%)		––	––
**Hospital Readmission within 30 days**				
Yes (N = 28)	7 (25.00%)	0.9710	0.983	0.364–2.395
No (N = 155)	39 (25.16%)		Ref	Ref
Unknown (N = 0)	0 (0.00%)		––	––
	**Magnesium (n = 57)**			
**Length of Stay (days)**				
	––	0.6770	0.955	0.766–1.188
**Death within 90 days**				
Yes (N = 23)	5 (21.74%)		0.566	0.179–1.510
No (N = 159)	52 (32.70%)	0.2811	Ref	Ref
Unknown (N = 1)	0 (0.00%)		––	––
**Hospital Readmission within 30 days**				
Yes (N = 28)	9 (32.14%)	0.9186	1.046	0.424–2.426
No (N = 155)	48 (30.97%)		Ref	Ref
Unknown (N = 0)	0 (0.00%)		––	––

N is the number of participants per row, and n is the number of participants with a deficiency per category. C.I. = confidence interval. Missing data were excluded from analyses due to ≤ 10% of the sample composition. Negative binomial regression tested the association between length of stay and micronutrient deficiencies, adjusted for age and birth sex. Incidence rate ratios (IRRs) and 95% CI were generated from the negative binomial regression for length of stay associations. Chi-square tested associations between remaining categorical variables and each of the micronutrient deficiencies, with *p* ≤ 0.05 considered significant. Logistic regression generated odds ratios and 95% confidence intervals. Cyanocobalamin and folic acid were omitted due to low counts. Reference groups (Ref) for each micronutrient were not deficient/normal. Double dashes indicate analyses excluded missing values due to low counts (n ≤ 2; ≤10% total sample).

**Table 5:** Relationship between thiamine deficiency and any alcohol consumption (n = 183).

	Thiamine Deficiency (n = 49)			
	n (%)	*p*-Value	Odds Ratio	95% Confidence Interval
**Any Alcohol Consumption**				
Yes (N = 48)	13 (27.08%)	0.8335	1.083	0.501–2.252
No (N = 135)	36 (26.67%)		Ref	Ref
Unknown (N = 0)	0 (0.00%)		––	––
**Alcohol Consumption Level**				
Moderate (N = 20)	5 (25.00%)	0.8893	0.545	0.063–5.303
Mild (N = 28)	8 (28.57%)		0.889	0.141–7.364
None (N = 135)	36 (26.67%)		Ref	Ref
Unknown (N = 0)	0 (0.00%)		––	––

N is the number of participants per alcohol consumption category, and n is the number with thiamine deficiency. Chi-square analysis tested associations, with *p* ≤ 0.05 considered significant. Logistic regression produced odds ratios with significance assessed using 95% confidence intervals. The reference group (Ref) was no alcohol consumption. Double dashes indicate analyses excluded missing values due to low counts (n ≤ 2; ≤10% total sample).

**Table 6:** Frequency of symptoms (N = 48) and clinical syndromes of thiamine deficiency for participants with low plasma thiamine.

TD Symptoms (N = 48 Surveys)	n/N (%)
Loss of appetite	42/48 (87.50%)
Loss of balance	30/48 (62.50%)
Nausea or vomiting	29/48 (60.42%)
Constipation	28/48 (58.33%)
Weakness or falls	26/48 (54.17%)
Confusion or trouble thinking	23/48 (47.92%)
Memory loss	17/48 (35.42%)
Double vision	6/48 (12.50%)
No symptoms	4/48 (8.33%)
Hallucinations	3/48 (6.25%)
Seizure	0/48 (0.00%)
TD Syndromes (N = 43 exams)	
Wernicke’s encephalopathy	26/43 (60.47%)
Gastrointestinal beriberi *	29/48 * (60.42%)
Dry beriberi	25/43 (58.14%)
Wet beriberi	3/43 (6.98%)
No TD syndrome	4/43 (9.30%)
One TD syndrome	11/43 (25.58%)
Two TD syndromes	16/43 (37.21%)
Three TD syndromes	9/43 (20.93%)
Four TD syndromes	3/43 (6.98%)
No symptoms or syndromes	1/48 (2.08%)
No survey or exam completed	1/49 (2.04%)

For symptoms, the total sample size is 48, with one subject missing an interview out of 49 participants with low plasma thiamine. For physical findings, the total sample size is 43, with 6 subjects missing physical examination. N is the number of subjects with results in the category, and n is the number of subjects with specific symptoms or findings. * Gastrointestinal beriberi was determined from symptoms, N = 48.

## Data Availability

Data that support the findings of this study are available on request from the corresponding author. The data are not publicly available due to privacy or ethical restrictions.

## Abbreviations

The following abbreviations are used in this manuscript:

AUD	alcohol use disorder
BMI	body mass index
CBC	complete blood count
CI	confidence interval
CRP	C-reactive protein
DRI	Dietary Reference Intake
ESRD	end-stage renal disease
ETKA	erythrocyte transketolase assay
GI	gastrointestinal
GLIM	Global Leadership Initiative on Malnutrition
Hg	hemoglobin
hs-CRP	highly sensitive C-reactive protein
IF	intrinsic factor
IRR	incidence rate ratio
LC-MS	liquid chromatography/mass spectroscopy
LMIC	low- and middle-income countries
LOS	lengths of hospital stay
MCV	mean corpuscular volume
MiDs	micronutrient deficiencies
MOCA	Montreal Cognitive Assessment
OR	odds ratio
RBCs	red blood cells
TD	thiamine deficiency
TDDs	thiamine deficiency disorders
TDP	thiamine diphosphate
U.S.	United States
VA	Veterans Administration
VASNHCS	VA Sierra Nevada Healthcare System
WE	Wernicke’s encephalopathy
25(OH)D	25-hydroxyvitamin D

## References

[1] Berger M.M., Pantet O., Schneider A., Ben-Hamouda N. (2019). Micronutrient Deficiencies in Medical and Surgical Inpatients. J. Clin. Med..

[2] Hoffman R.S., Weinhouse G.L. ‘Management of Moderate and Severe Alcohol Withdrawal Syndromes’, UpToDate. https://www.uptodate.com/contents/management-of-moderate-and-severe-alcohol-withdrawal-syndromes?search=Managementofmoderateandseverealcoholwithdrawalsyndromes&source=search_result&selectedTitle=1~150&usage_type=default&display_rank=1.

[3] Sarkisian C.A., Lachs M.S. (1996). “Failure to thrive” in older adults. Ann. Intern. Med..

[4] Butterworth R.F., Shils M., Shike M., Ross A., Caballero B., Cousins R. (2006). Thiamin. Modern Nutrition in Health and Disease.

[5] Berger M.M., Shenkin A., Schweinlin A., Amrein K., Augsburger M., Biesalski H.-K., Bischoff S.C., Casaer M.P., Gundogan K., Lepp H.-L. (2022). ESPEN micronutrient guideline. Clin. Nutr..

[6] Donnino M.W., Carney E., Cocchi M.N., Barbash I., Chase M., Joyce N., Chou P.P., Ngo L. (2010). Thiamine deficiency in critically ill patients with sepsis. J. Crit. Care.

[7] Lough M.E. (2012). Wernicke’s encephalopathy: Expanding the diagnostic toolbox. Neuropsychol. Rev..

[8] Caine D., Halliday G.M., Kril J.J., Harper C.G. (1997). Operational criteria for the classification of chronic alcoholics: Identification of Wernicke’s encephalopathy. J. Neurol. Neurosurg. Psychiatry.

[9] Sechi G., Serra A. (2007). Wernicke’s encephalopathy: New clinical settings and recent advances in diagnosis and management. Lancet Neurol..

[10] Nisar S., Tanvir M., Ganie M.A., Pharm O.K.M., Muzaffer U., Wani I.A. (2022). Clinical profile of patients presenting with thiamine-responsive upper-gastrointestinal upset: A pointer toward gastric beriberi. Nutrition.

[11] Smith T.J., Johnson C.R., Koshy R., Hess S.Y., Qureshi U.A., Mynak M.L., Fischer P.R. (2021). Thiamine Deficiency Disorders: A Clinical Perspective. Ann. N. Y. Acad. Sci..

[12] Whitfield K.C., Bourassa M.W., Adamolekun B., Bergeron G., Bettendorff L., Brown K.H., Cox L., Fattal-Valevski A., Fischer P.R., Frank E.L. (2018). Thiamine deficiency disorders: Diagnosis, prevalence, and a roadmap for global control programs. Ann. N. Y. Acad. Sci..

[13] Johnson C.R., Fischer P.R., Thacher T.D., Topazian M.D., Bourassa M.W., Combs G.F. (2019). Thiamin deficiency in low- and middle-income countries: Disorders, prevalences, previous interventions and current recommendations. Nutr. Health.

[14] Dizdar O.S., Yıldız A., Gul C.B., Gunal A.I., Ersoy A., Gundogan K. (2020). The Effect of Hemodialysis, Peritoneal Dialysis and Renal Transplantation on Nutritional Status and Serum Micronutrient Levels in Patients with End-stage Renal Disease; Multicenter, 6-month Period, Longitudinal Study. J. Trace Elem. Med. Biol..

[15] Isenberg-Grzeda E., Rahane S., DeRosa A.P., Ellis J., Nicolson S.E. (2016). Wernicke-Korsakoff syndrome in patients with cancer: A systematic review. Lancet Oncol..

[16] Abou-Hashem R.M., Maamoun M.M.A., Hamza S.A., Fahmy H.M., Mortagy A.K. (2009). Thiamine Level in Hospitalized Elderly Egyptian Patients with Congestive Heart Failure and Left Ventricular Systolic Dysfunction. J. Am. Geriatr. Soc..

[17] Gold M., Chen M., Johnson K. (1995). Plasma and Red Blood Cell Thiamine Deficiency in Patients with Dementia of the Alzheimer’s Type. Arch. Neurol..

[18] Lin S., Leppla I.E., Yan H., Probert J.M., Randhawa P.A., Leoutsakos J.-M.S., Probasco J.C., Neufeld K.J. (2020). Prevalence and Improvement of Caine-Positive Wernicke-Korsakoff Syndrome in Psychiatric Inpatient Admissions. Psychosomatics.

[19] Ehsanian R., Anderson S., Schneider B., Kennedy D., Mansourian V. (2020). Prevalence of Low Plasma Vitamin B1 in the Stroke Population Admitted to Acute Inpatient Rehabilitation. Nutrients.

[20] Moskowitz A., Graver A., Giberson T., Berg K., Liu X., Uber A., Gautam S., Donnino M.W. (2014). The Relationship Between Lactate and Thiamine Levels in Patients with Diabetic Ketoacidosis. J. Crit. Care.

[21] Attaluri P., Castillo A., Edriss H., Nugent K. (2018). Thiamine Deficiency: An Important Consideration in Critically Ill Patients. Am. J. Med. Sci..

[22] Nath A., Tran T., Shope T.R., Koch T.R. (2017). Prevalence of Clinical Thiamine Deficiency in Individuals with Medically Complicated Obesity. Nutr. Res..

[23] Fiorentini D., Cappadone C., Farruggia G., Prata C. (2021). Magnesium: Biochemistry, Nutrition, Detection, and Social Impact of Diseases Linked to its Deficiency. Nutrients.

[24] Touyz R.M., de Baaij J.H.F., Hoenderop J.G.J. (2024). Magnesium disorders. N. Engl. J. Med..

[25] Nielsen F.H., Johnson L.A.K. (2017). Data from Controlled Metabolic Ward Studies Provide Guidance for the Determination of Status Indicators and Dietary Requirements for Magnesium. Biol. Trace Elem. Res..

[26] Rubeiz G.J., Thill-Baharozian M., Hardie D., Carlson R.W. (1993). Association of Hypomagnesemia and Mortality in Acutely Ill Medical Patients. Crit. Care Med..

[27] Workinger J.L., Doyle R.P., Bortz J. (2018). Challenges in the Diagnosis of Magnesium Status. Nutrients.

[28] Gautam S., Khapung A. (2021). Prevalence of Hypomagnesemia Among Elderly Patients Attending a Tertiary Care Center: A Descriptive Cross-sectional Study. J. Nepal Med. Assoc..

[29] Malik D., Narayanasamy N., Pratyusha V.A., Thakur J., Sinha N. (2023). Fat-Soluble Vitamins. Textbook of Nutritional Biochemistry.

[30] Roth D.E., Abrams S.A., Aloia J., Bergeron G., Bourassa M.W., Brown K.H., Calvo M.S., Cashman K.D., Combs G., De-Regil L.M. (2018). Global prevalence and disease burden of vitamin D deficiency: A roadmap for action in low- and middle-income countries. Ann. N. Y. Acad. Sci..

[31] Belenchia A.M., Johnson S.A., Kieschnick A.C., Rosenfeld C.S., Peterson C.A. (2017). Time Course of Vitamin D Depletion and Repletion in Reproductive-age Female C57BL/6 Mice. Comp. Med..

[32] Duplessis C.A., Harris E.B., Watenpaugh D.E., Horn W.G. (2005). Vitamin D supplementation in underway submariners. Aviat. Space Environ. Med..

[33] Elrayah E.E., Rogers L., Doggui R., Al-Jawaldeh A. (2020). Vitamin D Insufficiency and Deficiency in the Eastern Mediterranean Region (EMR)-Misconceptions in Public Health Practice: A Scoping Review 2019–2020. J. Nutr. Sci. Vitaminol..

[34] Islam T., Peiris P., Copeland R.J., El Zoghby M., Peiris A.N. (2011). Vitamin D: Lessons from the Veterans Population. J. Am. Med. Dir. Assoc..

[35] Bailey L.B., Stover P.J., McNulty H., Fenech M.F., Gregory J.F., Mills J.L., Pfeiffer C.M., Fazili Z., Zhang M., Ueland P.M. (2015). Biomarkers of Nutrition for Development-Folate Review. J. Nutr..

[36] McLean E., de Benoist B., Allen L.H. (2008). Review of the magnitude of folate and vitamin B12 deficiencies worldwide. Food Nutr. Bull..

[37] Gudgeon P., Cavalcanti R. (2015). Folate Testing in Hospital Inpatients. Am. J. Med..

[38] Skorochod R., Shteingart S., Nesher G. (2021). Incidence of Inpatient Folate Deficiency and Effect on Overall In-Hospital Mortality. Am. J. Med..

[39] Carmel R., Shils M., Shike M., Catharine R.A., Caballero B., Cousins R.J. (2006). Cobalamin (Vitamin B12). Modern Nutrition in Health and Disease.

[40] Andrès E., Vidal-Alaball J., Federici L., Loukili N., Zimmer J., Kaltenbach G. (2007). Clinical aspects of cobalamin deficiency in elderly patients. Epidemiology, causes, clinical manifestations, and treatment with special focus on oral cobalamin therapy. Eur. J. Intern. Med..

[41] Berger M.M., Talwar D., Shenkin A. (2023). Pitfalls in the interpretation of blood tests used to assess and monitor micronutrient nutrition status. Nutr. Clin. Pract..

[42] Gabay C., Kushner I. (1999). Acute-phase proteins and other systemic responses to inflammation. N. Engl. J. Med..

[43] Han E., Fritzer-Szekeres M., Szekeres T., Gehrig T., Gyöngyösi M., Bergler-Klein J. (2022). Comparison of High-Sensitivity C-Reactive Protein vs C-reactive Protein for Cardiovascular Risk Prediction in Chronic Cardiac Disease. J. Appl. Lab. Med..

[44] Rodrigues L.P., Rezende A.T.d.O., Delpino F.M., Mendonça C.R., Noll M., Nunes B.P., de Oliviera C., Silveira E.A. (2022). Association between multimorbidity and hospitalization in older adults: Systematic review and meta-analysis. Age Ageing.

[45] Naik H., Murray T.M., Khan M., Daly-Grafstein D., Liu G., Kassen B.O., Onrot J., Sutherland J.M., Staples J.A. (2024). Population-Based Trends in Complexity of Hospital Inpatients. JAMA Intern. Med..

[46] Mates E., Alluri D., Artis T., Riddle M.S. (2021). A Retrospective Case Series of Thiamine Deficiency in Non-Alcoholic Hospitalized Veterans: An Important Cause of Delirium and Falling?. J. Clin. Med..

[47] Hamilton R.K.B., Phelan C.H., Chin N.A., Wyman M.F., Lambrou N., Cobb N., Kind A.J., Blazel H., Asthana S., Gleason C.E. (2020). The U-ARE Protocol: A Pragmatic Approach to Decisional Capacity Assessment for Clinical Research. J. Alzheimer’s Dis..

[48] Lemoine A., Le Devehat C., Codaccioni J.L., Monges A., Bermond P., Salkeld R.M. (1980). Vitamin B1, B2, B6, and C status in hospital inpatients. Am. J. Clin. Nutr..

[49] Jamieson C.P., Obeid O.A., Powell-Tuck J. (1999). The thiamin, riboflavin and pyridoxine status of patients on emergency admission to hospital. Clin. Nutr..

[50] Lee D.C., Chu J., Satz W., Silbergleit R. (2000). Low plasma thiamine levels in elder patients admitted through the emergency department. Acad. Emerg. Med..

[51] Epitools. https://epitools.ausvet.com.au/oneproportion?OneProportion%5Bproportion%5D=0.25&OneProportion%5Bprecision%5D=0.05&OneProportion%5Bconf%5D=0.95&OneProportion%5Bpopsize%5D=300.

[52] Cederholm T., Jensen G.L., Correia M.I.T.D., Gonzalez M.C., Fukushima R., Higashiguchi T., Baptista G., Barazzoni R., Blaauw R., Coats A.J. (2019). GLIM criteria for the diagnosis of malnutrition—A consensus report from the global clinical nutrition community. Clin Nutr.

[53] O’Keeffe S.T., Tormey W.P., Glasgow R., Lavan J.N. (1994). Thiamine deficiency in hospitalized elderly patients. Gerontology.

[54] Pepersack T., Garbusinski J., Robberecht J., Beyer I., Willems D., Fuss M. (1999). Clinical relevance of thiamine status amongst hospitalized elderly patients. Gerontology.

[55] Rosenthal M.D., Glew R.H. (2009). Medical Biochemistry Human Metabolism in Health and Disease.

[56] Ali M., Gubler C.J., Al Saleh J., Farsak F.A. (1987). A comparison of transketolase assay and transketolase and lactate dehydrogenase activity levels in whole blood and red cell hemolysates and in leukocytes. Comp Biochem Physiol B..

[57] Warnock L.G., Prudhomme C.R., Wagner C. (1978). The Determination of Thiamin Pyrophosphate in Blood and Other Tissues, and Its Correlation with Erythrocyte Transketolase Activity. J. Nutr..

[58] Chen M.F., Chen L.T., Gold M., Boyce H.W. (1996). Plasma and erythrocyte thiamin concentrations in geriatric outpatients. J. Am. Coll. Nutr..

[59] da Silva J.S.V., Seres D.S., Sabino K., Adams S.C., Berdahl G.J., Citty S.W., Cober M.P., Evans D.C., Greaves J.R., Gura K.M. (2020). ASPEN Consensus Recommendations for Refeeding Syndrome. Nutr. Clin. Pract..

[60] McLaughlin K., Joyal K., Lee S., Corrado M., Marquis K., Anger K., Szumita P. (2020). Safety of intravenous push thiamine administration at a tertiary academic medical center. J. Am. Pharm. Assoc..

[61] Edwards K.A., Randall E.A., Tu-Maung N., Sannino D.R., Feder S., Angert E.R., Kraft C.E. (2019). Periplasmic binding protein-based magnetic isolation and detection of thiamine in complex biological matrices. Talanta.

[62] Williams S., Heuberger R. (2016). Outcomes of Vitamin D supplementation in adults who are deficient and critically ill: A review of the literature. Am. J. Ther..

[63] Grädel L., Merker M., Mueller B., Schuetz P. (2016). Screening and Treatment of Vitamin D Deficiency on Hospital Admission: Is There a Benefit for Medical Inpatients?. Am. J. Med..

[64] Máčová L., Bičíková M. (2021). Vitamin D: Current Challenges between the Laboratory and Clinical Practice. Nutrients.

[65] Al Alawi A.M., Majoni S.W., Falhammar H. (2018). Magnesium and Human Health: Perspectives and Research Directions. Int. J. Endocrinol..

